# Supplementary material for: Exploring the interplay between colorectal cancer subtypes genomic variants and cellular morphology: A deep-learning approach
Source: PLoS One. 2024 Sep 10;19(9):e0309380. doi: 10.1371/journal.pone.0309380 (PMC11386451; doi:10.1371/journal.pone.0309380)
Supplement: Attachment — Submitted filename: ResponseToReviewer.pdf [file pone.0309380.s001.pdf]

Reply to the reviewers' comments for the paper:

Exploring the Interplay Between Colorectal Cancer Subtypes, Genomic Variants, and Cellular Morphology: A Deep-Learning Approach

Submitted to PLOS ONE

Manuscript Number: PONE-D-24-07948R1

Hadar Hezi, Daniel Shats, Daniel Gurevich, Yosef E. Maruvka, Moti Freiman

May 22, 2024

Dear Prof. Chenette, Editor in Chief, and Prof. Huang, Academic Editor,
PLOS ONE,

We thank the editor-in-chief, the academic editor, and the reviewer for their thorough and insightful comments. We have carefully considered each comment and have made the appropriate additions and changes in the paper to reflect them as described below.

Specifically, we made the following revisions to the manuscript:

1. We added a figure presenting WSI patches that 1) were classified incorrectly by the baseline model and correctly by our approach, and 2) were classified incorrectly by both the baseline model and our approach.

2. We included a discussion on cellular morphology in our results section.

Should you have any further questions, please do not hesitate to contact us.

Best regards,
The Authors

Reviewer 1 comments

1. **"This manuscript described a development of artificial intelligence (AI) for classification of subtype of colorectal cancer (CRC) using tumor tissue image of H&E stains. By incorporating genomic information for classification, the AI models performed higher accuracy than baseline AI models. This is novel and valuable. Authors**

showed the results based on statistical data."
We thank the reviewer for the positive feedback on our manuscript.

2. **"However, no example images were shown to account for the success of classification. The description would help scientists to recognize what structures in CRC image of microsatellite instability are important and are recognized by the AI models."**
We thank the reviewer for this comment. In response, we have added a new figure to the revised manuscript (Fig. 11) that illustrates examples of patches misclassified by the baseline model but correctly identified by our proposed model, along with patches that neither model classified accurately. The new figure has been included below for the convenience of the reviewer.

"Figure 11 depicts patches from patients that were classified incorrectly by the baseline model but correctly by our BP-CNN$_{\text{Combined}}$ model (top row) as well as patches from patients that were classified incorrectly by both models (bottom row)."

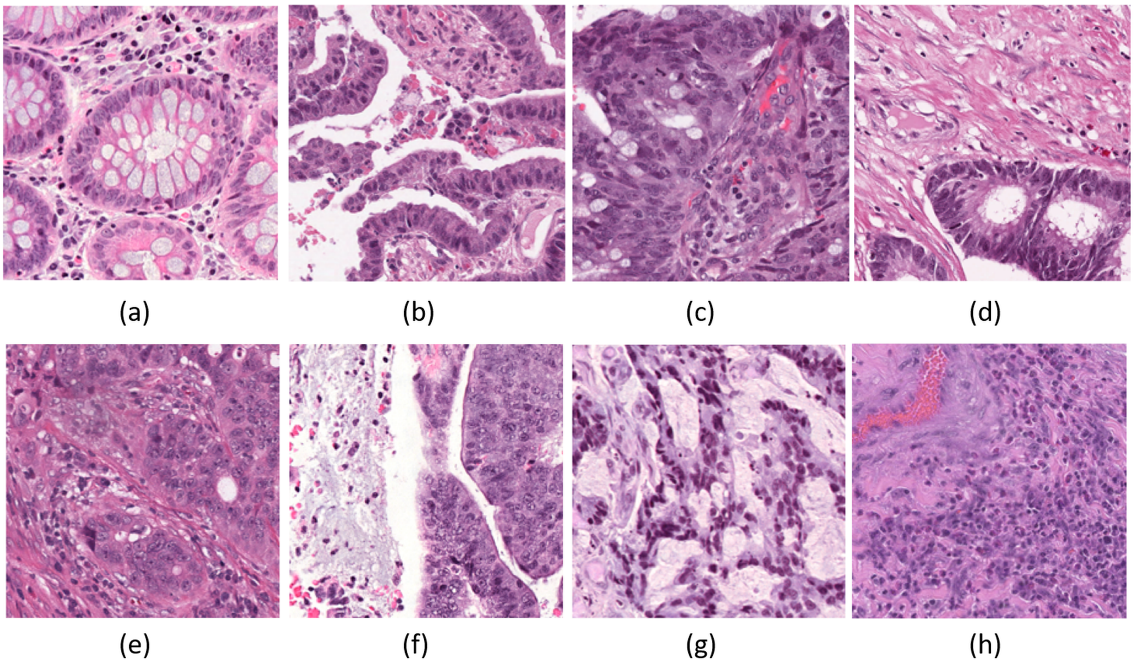

Figure 1: Patches of patients that were miss-classified by our models. Top row: patches of patients that were misclassified by the Baseline model and correctly classified by the BP-CNN$_{\text{Combined}}$ model. (a) TCGA-AA-3833, Baseline: MSS, BP-CNN$_{\text{Combined}}$: MSI, reference: MSI (SNP<1200), (b) TCGA-AY-6197, Baseline: MSS, BP-CNN$_{\text{Combined}}$: MSI, reference: MSI (CIMP-low), (c) TCGA-A6-2685, Baseline: MSI, BP-CNN$_{\text{Combined}}$: MSS, reference: MSS, (d) TCGA-NH-A6GC, Baseline: MSI, BP-CNN$_{\text{Combined}}$: MSS, reference: MSS. Bottom row: patches of patients that were misclassified by both the Baseline model and the BP-CNN$_{\text{Combined}}$ model. (e) TCGA-A6-2686, Baseline: MSS, BP-CNN$_{\text{Combined}}$: MSS, reference: MSI, (f) TCGA-AG-A02N, Baseline: MSS, BP-CNN$_{\text{Combined}}$: MSS, reference: MSI, (g) TCGA-AG-3881, Baseline: MSI, BP-CNN$_{\text{Combined}}$: MSI, reference: MSS, (h) TCGA-DC-6682, Baseline: MSI, BP-CNN$_{\text{Combined}}$: MSI, reference: MSS.

3. **"Authors would need to add figures on example of images which successfully recognized by the new AI model. Those images were failed to be classified appropriately by baseline AI models."**
   Thank you for your feedback. As noted earlier, we have included the requested figure in the revised version of the manuscript.

4. **"Comments on the cellular morphology would be interesting for readers."**
   We thank the reviewer for this comment. We have expanded our discussion on cellular morphology within the correctly and incorrectly classified patches in the results section of our manuscript. Specifically, we have added the following paragraphs: "Patches (a, b), correctly identified as MSI by our BP-$\text{CNN}_{\text{Combined}}$ model but mislabeled as MSS by the baseline, display pronounced nuclear pleomorphism and prominent nucleoli—hallmarks of MSI. This suggests that the BP-$\text{CNN}_{\text{Combined}}$ model is more adept at detecting these subtle yet crucial variations than the baseline model. Conversely, patches (c, d) that were correctly identified as MSS by our BP-$\text{CNN}_{\text{Combined}}$ model but misclassified as MSI by the baseline likely exhibit characteristics such as poor gland formation and high intra-tumoral lymphocytes, which are typically indicative of MSI, demonstrating that our BP-$\text{CNN}_{\text{Combined}}$ model can correctly interpret these complex features, where the baseline model fails.

   The patches misclassified by both models (bottom row) likely exhibit mixed features or subtle signs that pose classification challenges, including moderate gland formation with irregularities and characteristics that straddle the line between MSI and MSS, such as moderate nuclear pleomorphism and visible but subdued nucleoli. These ambiguous features contribute to the confusion in model classifications. "

5. **"Line 240. Please provide the full name of abbreviation CNV..."**
   We thank the reviewer for indicating this. We provided the full name of CNV on the first appearance (line 107 in the original submission) in the revised version. the updated line is: "The genomic information including the SNP rates, CIMP types, and Copy number variation (CNV) values was provided by Liu et al."
