# Supplementary material for: Exploring the interplay between colorectal cancer subtypes genomic variants and cellular morphology: A deep-learning approach
Source: PLoS One. 2024 Sep 10;19(9):e0309380. doi: 10.1371/journal.pone.0309380 (PMC11386451; doi:10.1371/journal.pone.0309380)
Supplement: Attachment — Submitted filename: Response_to_the_reviewers.pdf [file pone.0309380.s002.pdf]

Reply to the reviewers' comments for the paper:

Exploring the Interplay Between Colorectal Cancer Subtypes, Genomic Variants, and Cellular Morphology: A Deep-Learning Approach

Submitted to PLOS ONE

Manuscript Number: PONE-D-24-07948R1

Hadar Hezi, Daniel Shats, Daniel Gurevich, Yosef E. Maruvka, Moti Freiman

June 30, 2024

Dear Prof. Chenette, Editor in Chief, and Prof. Huang, Academic Editor,
PLOS ONE,

We thank the editor-in-chief, the academic editor, and the reviewer for their thorough and insightful comments. We have carefully considered each comment and have made the appropriate additions and changes in the paper to reflect them as described below. Should you have any further questions, please do not hesitate to contact us.

Best regards,
The Authors

Reviewer 1 comments

1. **"Please add a description on what is referred by the positive in Fig 4. MSI? It is not clear."**
   We apologize for this unclear description. In response to the reviewer's comment, we added the description of what is referred to by the positive in Fig 4. in the "genomic variations analysis" sub-section in the Results section. Specifically, the following paragraph was added: "Figure 4 presents three molecular features of our CRC patients at the patient level, plotted against the patch classification from our baseline model. In this figure, the MSI class is denoted as the positive class, and the MSS class is denoted as the negative class."

2. **"Please add brief description on CIMP-H in section "Genomic variations analysis". What is referred by H. Biological explanation would**

be needed for better understanding of your research hypothesis."
We thank the reviewer for this comment. In response, we have added a brief description on CIMP-H in the section "Genomic variations analysis". Specifically, the following paragraph was added: "Drawing from Liu et al. findings [17], SNP mutations are highly frequent in MSI patients due to their deficiency in the DNA mismatch-repair mechanism. However, MSI samples exhibit significant disparities in SNP density, ranging dramatically from 10 to 17,000, with a median of 1,432. Additionally, the CIMP rate, which influences gene silencing [24], is typically high in MSI patients. Specifically, 60% of MSI patches are categorized as CIMP-High (CIMP-H), while the remaining 40% are non-CIMP-H and categorized as CIMP-low and non-CIMP."

3. **"A calculation formula or explanation need to be shown on the "Non-CIMP-H patches in MSI (76%)" in section "Genomic variations analysis". In the previous sentence, it was described as "accounting for 59% of MSI patches". So, I assumed 100 − 59 = 41. Your description was 76."**
We apologize for the unclear description. We revised the text to better clarify that these percentages are associated with different aspects and do not cover two parts of the group. Therefore no need to sum up to 100%. Specifically, CIMP-H accounts for 59% of the MSI patches. From the rest 41% which are MSI but not CIMP-H, a substantial portion (76%) was incorrectly classified as MSS (negative class). We added the following paragraph to the text: "Highly methylated (CIMP-H) samples are rare in MSS, present in only 1% of the MSS patches, but are prevalent in MSI, accounting for 59% of the MSI patches. Notably, among the MSI patches that are non-CIMP-H, a substantial portion (76%) was incorrectly classified as MSS (negative class)."
