# Supplementary material for: Exploring the interplay between colorectal cancer subtypes genomic variants and cellular morphology: A deep-learning approach
Source: PLoS One. 2024 Sep 10;19(9):e0309380. doi: 10.1371/journal.pone.0309380 (PMC11386451; doi:10.1371/journal.pone.0309380)
Supplement: Attachment — Submitted filename: Response.pdf [file pone.0309380.s003.pdf]

Reply to the reviewers' comments for the paper:

# Exploring the Interplay Between Colorectal Cancer Subtypes, Genomic Variants, and Cellular Morphology: A Deep-Learning Approach

Submitted to PLOS ONE

Manuscript Number: PONE-D-24-07948R2

Hadar Hezi, Daniel Shats, Daniel Gurevich, Yosef E. Maruvka, Moti Freiman

July 25, 2024

Dear Prof. Chenette, Editor in Chief, and Prof. Huang, Academic Editor,
PLOS ONE,

We thank the editor-in-chief, the academic editor, and the reviewer for their thorough and insightful comments. We have carefully considered each comment and have made the appropriate additions and changes in the paper to reflect them as described below. Should you have any further questions, please do not hesitate to contact us.

Best regards,
The Authors

## Reviewer 1 comments

1. **"Thank you for the opportunity to review your interesting works. I would recommend your manuscript to be published in PLOS ONE."** Thank you very much for your kind words and for taking the time to review our work. We are delighted you find our manuscript interesting and appreciate your recommendation for publication in PLOS ONE.

## Reviewer 2 comments

1. **"The authors have picked a fascinating topic i.e, genomic and image data for subtype classification and to study their relationship using AI."**

We thank the reviewers for the kind feedback. We are pleased to hear that you find our topic on using AI for genomic and image data in subtype classification and their relationship fascinating.

2. **"Why did writers refer to their model as a 'Biologically-primed model'?"**
We thank the reviewer for raising this issue. The main difference between our proposed models and those previously proposed for CRC subtype classification lies in how they handle genomic variations within each subtype. Previous models consider only the CRC subtypes as potential classes, ignoring the heterogeneity within each subtype. In contrast, our model architecture accounts for this genomic heterogeneity, allowing for a more nuanced and accurate classification. To better reflect this, we term our model "biologically-primed," as it integrates biological variations within subtypes, leading to a more comprehensive and precise understanding of CRC subtypes.

   To better clarify that, we added the following explanation to our introduction: "We examined this hypothesis by developing and evaluating "biologically-primed" CNN classification models that account for the potential correlation between the genomic variations and the imaging phenotype. This is in contrast to previously proposed models for CRC subtype classification which considered only the CRC subtypes as potential classes, ignoring the heterogeneity within each subtype. To better reflect this, we term our model "biologically-primed," as it integrates biological variations within subtypes, leading to a more comprehensive and precise understanding of CRC subtypes."

3. **""consistent distribution of the genomic variations across each fold". Are you categorizing subtypes of Colorectal Cancer or examining genetic variations?"**
We thank the reviewer for pointing out this ambiguity. Our models aim to categorize CRC subtypes (i.e., MSI and MSS). However, recognizing the genomic heterogeneity within each subtype, the model architecture is designed to internally classify each subtype based on its genomic variations. Therefore, we ensured that the distribution of both the CRC subtypes and their internal genomic variations is consistent across the different folds. To better reflect this, we have revised the sentence mentioned by the reviewer as follows: "consistent distribution of the CRC subtypes and their internal genomic variations across each fold"

4. **"The elucidation of model is presently ambiguous. It would be advantageous if the authors could furnish a flow diagram that delineates the complete process, encompassing data preprocessing to model validation."**
We thank the reviewer for pointing this out. In response we included a new figure (Fig. 3 in the current revision) delineating the complete process, encompassing data preprocessing to model validation. The figure is enclosed below.

5. **"What is the number of SNPs or CpG sites used for model devel-**

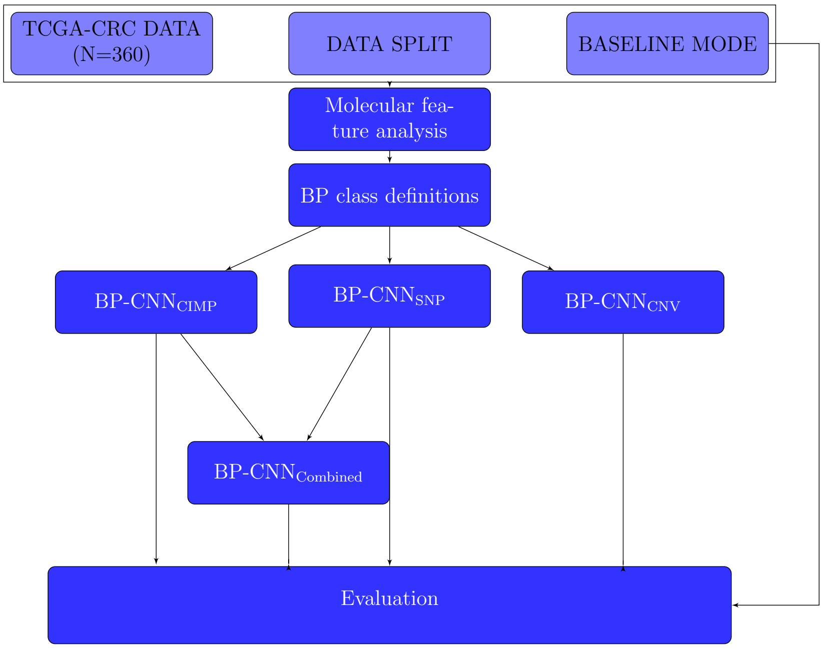


Figure 1: Experimental flow for our exploration of the interplay between CRC subtypes genomic variants and cellular morphology. The TCGA-CRC dataset, preprocessed by Kather et al. [7] (N=360) is split into different sets for analysis. A baseline model is trained, and based on its results, a molecular feature analysis is performed. Based on the analysis we choose to define our data classes based on the ranges and categories of SNP, CIMP and CNV (the BP class definitions step). After the definition, we divide the classes into five stratified folds. Next, three models are trained: BP-CNN$_{CIMP}$, BP-CNN$_{SNP}$, and BP-CNN$_{CNV}$ to evaluate the interplay between genomic variations and cellular morphology. The BP-CNN$_{CIMP}$, BP-CNN$_{SNP}$, and BP-CNN$_{CNV}$ models classify the data based on CIMP, SNP, and CNV features, respectively. Based on their results, BP-CNN$_{CIMP}$ and BP-CNN$_{SNP}$ are further combined into BP-CNN$_{Combined}$ to incorporate the entire set of genomic variations identified as influencing cellular morphology.

**opment?"**
The number of SNPs and the CIMP category are extracted from a patient's DNA sample in the TCGA. The range of SNPs among patients varied from 20 to 5000. Based on our analysis described in the Results / Genomic Variations Analysis section, we set the SNP threshold to 1200 and divided the CIMP categories into CIMP-H and non-CIMP-H. To better clarify this aspect, we added the following details to our Results / Genomic Variations Analysis section:

"The number of SNPs and the CIMP category are extracted from a patient's DNA sample in the TCGA. The range of SNPs among patients varied from 20 to 5000."

6. **'The authors have not provided a clear explanation of how they have combined the genomic and imaging data"**
We thank the reviewer for pointing this out. As shown in Fig. 2b in the manuscript, our model does not directly use genomic data as input. Instead, we represent the MSI class as two distinct classes based on their genomic variations. The genomic variation information is utilized during model training to label the MSI patches as either $MSI_1$ or $MSI_2$. To better clarify this aspect we added the following to our introduction: "It is important to highlight that our model does not directly use genomic data as input. Instead, we represent the CRC subtype class as two distinct classes based on their genomic variations. The genomic variation information is utilized during model training to label the MSI patches as either $MSI_1$ or $MSI_2$. Therefore, while our training phase incorporates molecular subtype information, the inference process depends exclusively on the H&E images, with no additional data used. "
